# MHD Mixed Convection and Entropy Generation in a Lid-Driven Triangular Cavity for Various Electrical Conductivity Models

**DOI:** 10.3390/e20120903

**Published:** 2018-11-25

**Authors:** Ali J. Chamkha, Fatih Selimefendigil, Hakan F. Oztop

**Affiliations:** 1 Mechanical Engineering Department, Prince Sultan Endowment for Energy and Environment, Prince Mohammad Bin Fahd University, Al-Khobar 31952, Saudi Arabia; 2 RAK Research and Innovation Center, American University of Ras Al Khaimah, Ras Al Khaimah 10021, UAE; 3 Department of Mechanical Engineering, Celal Bayar University, Manisa 45140, Turkey; 4 Department of Mechanical Engineering, Technology Faculty, Fırat University, Elazığ 23119, Turkey

**Keywords:** electrical conductivity, nanofluids, lid driven, triangular cavity, finite element, magneto-hydrodynamic (MHD)

## Abstract

In this study, effects of different electrical conductivity models for magneto- hydrodynamic mixed convection of nanofluids in a lid-driven triangular cavity was numerically investigated with a finite element method. Effects of Richardson number and Hartmann number on the convective heat transfer characteristics were analyzed for various electrical conductivity models of nanofluids. Average Nusselt number decreases for higher Hartmann and Richardson numbers. Discrepancies in the local and average heat transfer exist between different electrical conductivity models, which is higher for higher values of Richardson number and Hartmann number. The total entropy generation rate was found reduced with higher values of Richardson number and Hartmann number while discrepancies exist between various electrical conductivity models. When the magnetic field is imposed, different behaviors of entropy generation rate versus solid particle volume fraction curve is obtained and it is dependent upon the range of solid particle volume fraction.

## 1. Introduction

Mixed convection is important for a variety of thermal engineering applications ranging from electronic cooling to solar power [1,2]. The interactions between the shear driven flow and natural convective effects complicated the analysis of mixed convection [3,4,5]. In various engineering applications, the configurations can be simplified to convection in cavity of different geometrical shapes such as square, trapezoidal and triangular cavity. In this study, a triangular cavity with a partial heater located at the bottom wall is considered. Convection in triangular cavities is of importance in some practical applications such as in the building roof, electronic devices and solar power [6,7]. In most of the studies, convection due to horizontal or vertical isothermal walls was considered [8], but, in some applications, partial heating or cooling is important such as in electronic cooling applications [9,10].

Effects of magnetic field are relevant to various technological applications such as in micro-electro-mechanical systems (MEMs), nuclear reactor coolers, and purification of molten metals. An external magnetic field could be used to control the convective heat transfer [11,12,13,14]. In the application of magnetic field within cavities, the magnetic field was found to dampen the fluid motion and reduced the convection [15,16]. In separated flow configurations, magnetic field has the potential to enhance the heat transfer [17,18]. Recently, in heat transfer applications, nanofluids have been extensively used [19,20,21]. These fluids are composed of a base fluid such as water, ethylene glycol and added ultra-metallic or non-metallic fine solid particles which have an average particle size less than 100 nm. Higher conductivity of the solid nanoparticles makes them attractive for heat transfer applications since thermal conductivity of the solid particle is much higher than that of the base fluid. Theoretical and experimental methods may be utilized for the description of nanofluid effective thermopysical properties. There are some advanced methods such as fractal method and Monte Carlo simulation method that could be used for the analysis of transport properties of porous nanofibers [22,23].

Magnetic field with nanofluid offers some advantages due to the higher electrical conductivity of the solid particles. In the modeling of magneto-hydrodynamic (MHD) convective heat transfer problems, among the other effective thermophysical properties of nanofluid, electrical conductivity modeling is also important. The Maxwell model [24] is the most widely used in MHD flow applications. There are some other electrical conductivity models that can be used for nanofluids. An experimental study for the electrical conductivity measurement of alumina-water nanofluid with 12 nm diameter particles was conducted in Ref. [25]. It was observed that electrical conductivity increases with nanoparticle volume fraction and temperature. In the study of Ref. [26], electrical conductivity of water-alumina nanofluid was given as a function of temperature and nanopartricle volume fraction. The effects of solid volume fraction were found to be significant when they were compared to dependency with temperature. In the experimental study of Shoghl et al. [27], various effective properties including the electrical conductivity of water based nanofluid were determined. It was noted that electrical conductivity of the nanofluid was strongly influenced by the inclusion of the nanoparticles. In a recent study, Selimefendigil and Oztop [28], MHD mixed convection of nanofluid in a trapezoidal cavity was performed with various electrical conductivity models of nanofluid. An optimization study was also performed and it was noted that, depending on the electrical conductivity model of nanofluid, the optimum value of magnetic inclination angle changes. Significant changes in the heat transfer rate between different electrical conductivity models were also observed. Karimipour et al. [29] numerically studied the forced convection in a micro-channel with magnetics for two different nanoparticles. It was observed that nanoparticle with higher thermal conductivity was beneficial for heat transfer enhancement when the Reynolds number is higher.

Recently, in thermal engineering analysis of engineering problems, entropy generation analysis was also included [30,31]. This analysis can be used for system performance evaluation under different operating conditions [32,33]. The irreversibility due to the heat transfer and fluid friction can be quantified and can be included in the analysis. Bejan [34] presented the fundamentals of entropy generation minimization. A comparison of entropy generation analysis for single and two-phase modeling approach of nanofluid was performed for turbulent flow in a horizontal tube in Ref. [35]. Three different two-phase model approaches were utilized. Discrepancies between the models were observed for higher nanoparticle volume fraction. There are many studies that consider the entropy generation and second law analysis for nanofluids under the effect of magnetic field [36,37].

In this study, we numerically examined the magneto-hydro dynamic (MHD) mixed convection and entropy generation of water-alumina nanofluid in a lid-driven triangular cavity with partial heater for various electrical conductivity models. The results of this investigation can be used for design and optimization of convection in triangular cavities where a lot of application areas exist as mentioned above. In the literature, a vast amount of studies are dedicated to the application of convective heat transfer with nanofluids under the effect of magnetic field. In most of these studies, the Maxwell model for the electrical conductivity of the nanofluid was utilized. However, various electrical conductivity models may have significant impact on the fluid flow and heat transfer features. In this study, second law analysis for various electrical conductivity models on the entropy generation is also considered. The numerical simulation results are expressed with streamline, isotherm plots and local and average Nusselt number distribution plots for various values of Richardson and Hartmann numbers considering three different electrical conductivity models.

## 2. Numerical Modeling

**Figure 1:**
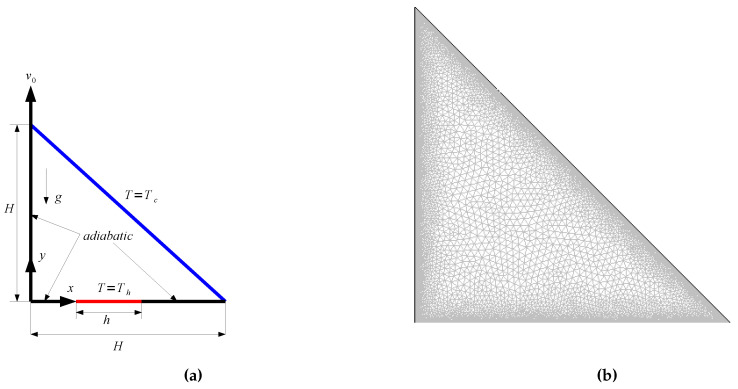
Schematic description of the physical model and boundary conditions (**a**) and mesh distribution of computational domain (**b**).

Figure 1 shows a schematic representation of the model problem. A lid-driven triangular cavity filled with alumina-water nanofluid was considered. The left vertical wall is moving in the +y direction with velocity of $v_{0}$. The bottom wall is partly kept at constant temperature of T*_h_* while the inclined wall is at a temperature of T*_c_* (T*_h_* > T*_c_*). The size of the heater is h=0.5H while it is located at 0.25H≤x≤0.75H. Alumina-water nanofluid with different solid nanoparticle volume fractions was used. Table 1 shows the thermophysical properties of water and alumina nanoparticle. The gravitational acceleration is in the negative *y*-direction. Boussinesq approximation was used for modeling the density change in the buoyancy term. A uniform magnetic field was utilized which makes an angle of 45 degrees with the horizontal. Various effects including joule heating, induced magnetic field and displacement currents are assumed to be negligible. Thermal radiation and viscous dissipation effects are also neglected.

Conservation equations of fluid flow and heat transfer are written as follows [38]:

(1) $$\frac{\partial u}{\partial x}+\frac{\partial v}{\partial y}=0,$$

(2) $$\begin{array}{c} u\frac{\partial u}{\partial x}+v\frac{\partial u}{\partial y}=-\frac{1}{\rho_{\mathrm{nf}}}\frac{\partial p}{\partial x}+\nu_{\mathrm{nf}}\left(\frac{\partial^{2}u}{\partial x^{2}}+\frac{\partial^{2}u}{\partial y^{2}}\right)\\ +\frac{\sigma_{nf}B_{0}^{2}}{\rho_{nf}}\left(v\sin\left(\gamma \right)\cos\left(\gamma \right)-u\sin^{2}\left(\gamma \right)\right), \end{array}$$

(3) $$\begin{array}{c} u\frac{\partial v}{\partial x}+v\frac{\partial v}{\partial y}=-\frac{1}{\rho_{\mathrm{nf}}}\frac{\partial p}{\partial y}+\nu_{\mathrm{nf}}\left(\frac{\partial^{2}v}{\partial x^{2}}+\frac{\partial^{2}v}{\partial y^{2}}\right)\\ +\beta_{\mathrm{nf}}g\left(T-T_{c}\right)+\frac{\sigma_{nf}B_{0}^{2}}{\rho_{nf}}\left(u\sin\left(\gamma \right)\cos\left(\gamma \right)-v\cos^{2}\left(\gamma \right)\right), \end{array}$$

(4) $$u\frac{\partial T}{\partial x}+v\frac{\partial T}{\partial y}=\alpha_{\mathrm{nf}}\left(\frac{\partial^{2}T}{\partial x^{2}}+\frac{\partial^{2}T}{\partial y^{2}}\right).$$

Entropy generation equation can be written as [38]:

(5) $$\begin{array}{c} S=\frac{k_{nf}}{T_{0}^{2}}\left[{\left(\frac{\partial T}{\partial x}\right)}^{2}+{\left(\frac{\partial T}{\partial y}\right)}^{2}\right]+\frac{\mu_{nf}}{T_{0}}\left[2\left({\left(\frac{\partial u}{\partial x}\right)}^{2}+{\left(\frac{\partial v}{\partial y}\right)}^{2}\right)+{\left(\frac{\partial u}{\partial x}+\frac{\partial v}{\partial y}\right)}^{2}\right]\\ +\frac{\sigma_{nf}B_{0}^{2}}{T_{0}}{\left(u\sin\gamma -v\cos\gamma \right)}^{2}. \end{array}$$

The contributions due to the various effects such as heat transfer, viscous dissipation and MHD are represented by various terms in the above equation.

Following non-dimensional parameters are used for converting the above equations in non-dimensional form [38]:

(6) X=xH,Y=yH,U=uu0,V=vu0,P=pρfu02,θ=T−TcTh−Tc,Gr=gβf(Th−Tc)H3νf2,Pr=νfαf,Ra=GrPr,Ha=B0Hσfμf,Re=u0Hνf,Ri=GrRe2.

The effective density, specific heat and thermal expansion coefficient are described as [3]:

(7) $$\rho_{nf}=\left(1-\phi \right)\rho_{bf}+\phi \rho_{p},$$

(8) $${\left(\rho c_{p}\right)}_{nf}=\left(1-\phi \right){\left(\rho c_{p}\right)}_{bf}+\phi {\left(\rho c_{p}\right)}_{p}.$$

The effective thermal expansion coefficient of the nanofluid is defined as:

(9) $${\left(\rho \beta \right)}_{nf}=\left(1-\phi \right){\left(\rho \beta \right)}_{bf}+\phi {\left(\rho \beta \right)}_{p}.$$

The effective thermal conductivity of the nanofluid is defined as follows [39]:

(10) $$\begin{array}{c} k_{nf}=k_{f}\left[\frac{\left(k_{p}+2k_{f}\right)-2\phi \left(k_{f}-k_{p}\right)}{\left(k_{p}+2k_{f}\right)+\phi \left(k_{f}-k_{p}\right)}\right]+5\times 10^{4}\phi \rho_{f}c_{p,f}\sqrt{\frac{\kappa_{b}T}{\rho_{p}d_{p}}}f^{\prime }\left(T,\phi ,d_{p}\right), \end{array}$$

and the function $f^{\prime }$ was given in [39]. The Brownian motion was included in the above definition. The effective viscosity is described as [39]:

(11) $$\mu_{nf}=\mu_{f}{\left(1-\phi \right)}^{-0.25}+\frac{k_{Brownian}}{k_{f}}\times \frac{\mu_{f}}{Pr_{f}}.$$

In the current study, various models for electrical conductivity of the alumina-water nanofluids were taken into account. Model 1 (M1) is the Maxwell’s model and the electrical conductivity is given as:

(12) $$\sigma_{nf}=\sigma_{f}\left(1+\frac{3(f-1)\phi }{(f+2)-(f-1)\phi }\right),$$

with $f=\frac{\sigma_{p}}{\sigma_{f}}$ denoting the conductivity ratio of solid and fluid phases. This model was derived for random suspension of spherical particles [24]. Model 2 (M2) was developed from experimental study as the effective electrical conductivity of alumina-water nanofluid in Ref. [26]. Effective electrical conductivity depends upon the nanoparticle volume fraction and temperature [26]:

(13) $$\sigma_{nf}=\sigma_{f}\left(3679.049\phi +1.085779T-42.6384\right).$$

Another electrical conductivity model of alumina-water nanofluid model was offered as Model 3 (M3) in Ref. [25]:

(14) $$\begin{array}{cc} \sigma_{nf} & =176.69+588.41\left(\phi \times 100\right)-13.64T-86.31{\left(\phi \times 100\right)}^{2}+0.36T^{2}+\\ & 1.07\left(\phi \times 100\right)T+11.06{\left(\phi \times 100\right)}^{3}-0.003T^{3}+0.18T^{2}\left(\phi \times 100\right)-1.01T{\left(\phi \times 100\right)}^{2}. \end{array}$$

The dimensional boundary conditions for the partially heated lid-driven triangular cavity can be written as follows:

- On the partial heater (part of bottom wall): u=v=0, T=T${}_{h}$,
- On the inclined wall: u=v=0, T=T${}_{v}$,
- On the adiabatic walls of bottom part: u=v=0, $\frac{\partial T}{\partial y}=0$,
- On the left vertical wall: u=0, v=v${}_{0}$, $\frac{\partial T}{\partial x}=0$.

Local and average Nusselt numbers for the hot wall are calculated as:

(15) $${\mathrm{Nu}}_{x}=-\frac{k_{nf}}{k_{f}}{\left(\frac{\partial \theta }{\partial Y}\right)}_{Y=0},\;\;\;{\mathrm{Nu}}_{m}=\frac{1}{h}\int_{0}^{h}{\mathrm{Nu}}_{x}dx,$$

with *h* representing the length of the heater.

Governing equations along with the boundary conditions as described in the previous subsection were solved by using the Galerkin weighted residual finite element method where weak form of the equations were obtained. For the approximation of the flow variables within the computational domain, Lagrange finite elements of different orders were utilized.The weighted residual *R* will be zero as:

(16) $$\int_{\Omega }w_{k}\left(x\right)Rdv=0,$$

with $w_{k}$ representing the weight function for which is chosen the same set of functions as the trial functions. Finally, nonlinear residual equations at the nodes of internal element domain are obtained and they were solved with the Newton–Raphson method.

**Figure 2:**
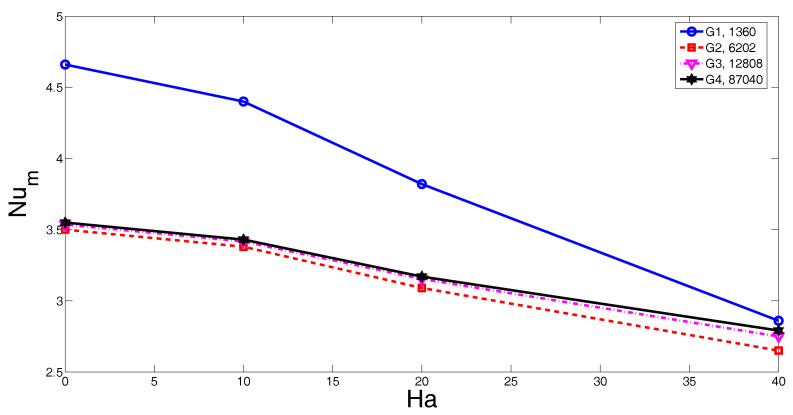
Grid independence test for various values of Hartmann numbers and grid sizes (Ri = 100, ϕ=0.01).

Various grid sizes are tested to obtain mesh independence of the solution. A grid distribution of the computational domain is demonstrated in Figure 1b. The grid is refined in the vicinity of the walls to resolve higher gradients of flow variables. Figure 2 shows the grid independence test results. The average Nusselt number versus Hartmann number plot is shown for various grid sizes (Ri = 1, ϕ=0.01). G3 with 12808 number of triangular elements is used in the subsequent computations. Validation of present solver was performed by using different existing works available in the literature. Numerical analysis results of Iwatsu et al. [40] were used where mixed convection in a lid-driven cavity was examined. Table 2 presents the average Nusselt number comparisons for various Grashof numbers at Reynolds number of 400. Another validation study was made by using the numerical results of Rudraiah et al. [41] where natural convection was examined under the effects of the magnetic field. Table 3 shows the comparison of the average Nusselt number for different Hartmann number when Grashof number is fixed to 2 × 10^4^. The numerical simulation results of Sheikholeslami and Shamlooei [42], which were calculated by using the lattice Boltzmann method, were also included in this table.

## 3. Results and Discussion

MHD mixed convection in a lid-driven triangular cavity filled with alumina-water nanofluid was numerically studied. Effects of Richardson number (between 0.01 and 100) and Hartmann number (between 0 and 40) on the convective heat transfer and fluid flow characteristics were examined. The Prandtl number of the base fluid is 6.9. Effects of three different electrical conductivity models on the mixed convective heat transfer were investigated. Second law analysis of the thermal configuration with entropy generation was also performed.

**Figure 3:**
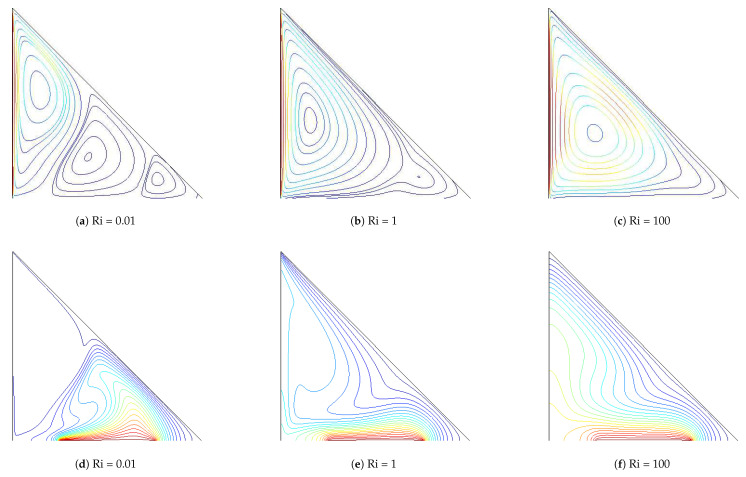
Streamline (**a–c**) and isotherm (**d–f**) distribution within the triangular cavity for different values of Richardson number (M1, Ha = 10, ϕ=0.02).

**Figure 4:**
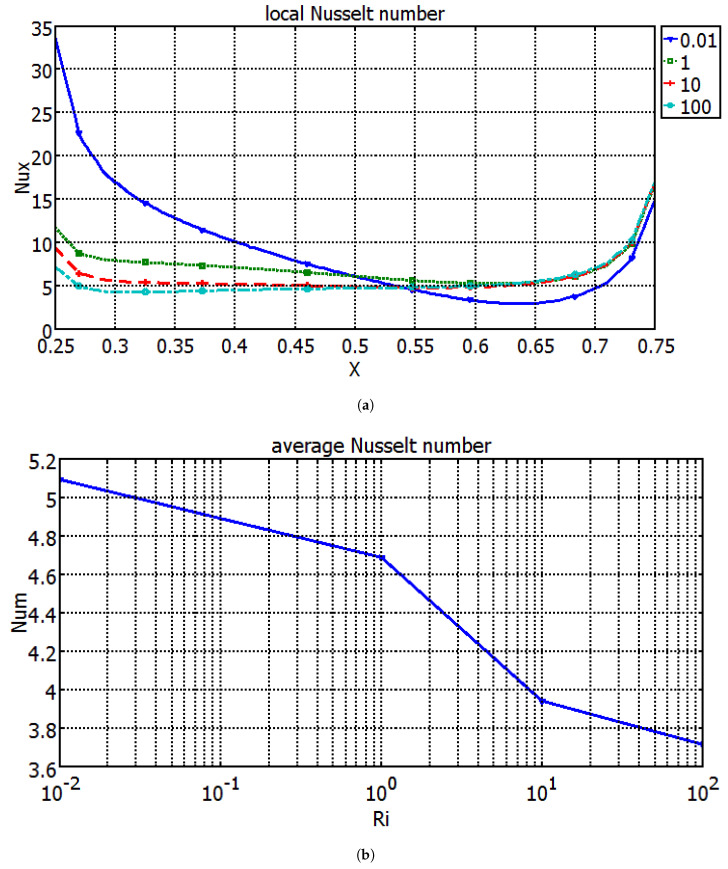
Variation of (**a**) local and (**b**) average Nusselt number along the hot wall for different Richardson numbers (M1, Ha = 10, ϕ=0.02).

Figure 3 demonstrates the distribution of streamline and isotherms within the triangular cavity for different values of Richardson number with Maxwell model (M1) at Hartmann number of 10. A lower value of Richardson number denotes a higher velocity of the moving wall as the Grashof number of the configuration is fixed. The triangular cavity is occupied with three recirculating zones for Richardson number of 0.01. As the wall velocity decreases, the natural convection effects become important and the number of vortices decreases for higher Richardson numbers. Temperature gradients are higher in the left part of the heater while isotherms become less dense for higher Richardson numbers, indicating less heat transfer process in those locations. Local and average Nusselt number reduce as the value of Richardson number enhances (Figure 4). At the lowest value of Richardson number, the local heat transfer becomes lower in the right part of the heater. The mixed convection studies within lid-driven triangular cavities show similar trends for the average heat transfer versus Richardson number variations when the relevant studies in the literature are examined. In the study of Ghasemi and Aminossadati [43], mixed convection within a lid-driven triangular cavity with nanofluid was examined numerically and it was observed that, as the value of Richardson number decreases, the average heat transfer increases. In the numerical study of Selimefendigil and Oztop [44], where mixed convection for a partially heated nanofluid-filled lid driven triangular enclosure with a flexible wall was examined, local and average Nusselt number were found to be reduced with the the rise of Richardson number.

**Figure 5:**
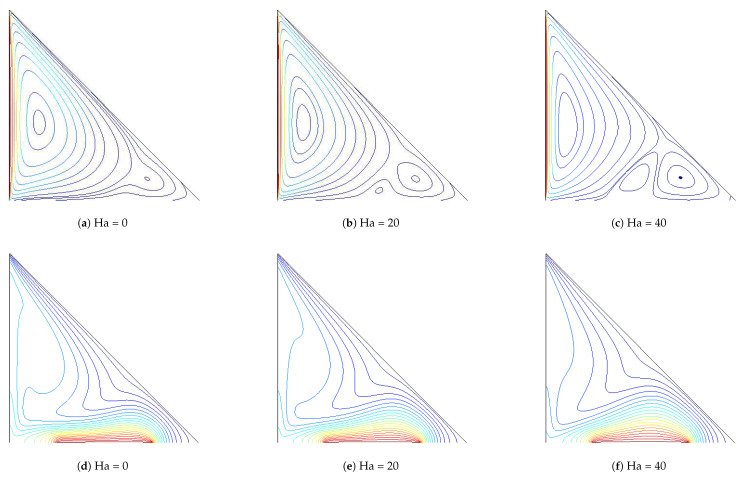
Effects of Hartmann number on the streamline (**a**–**c**) and isotherm (**d**–**f**) distribution within the triangular cavity with the M3 model (Ri = 1, ϕ=0.02).

**Figure 6:**
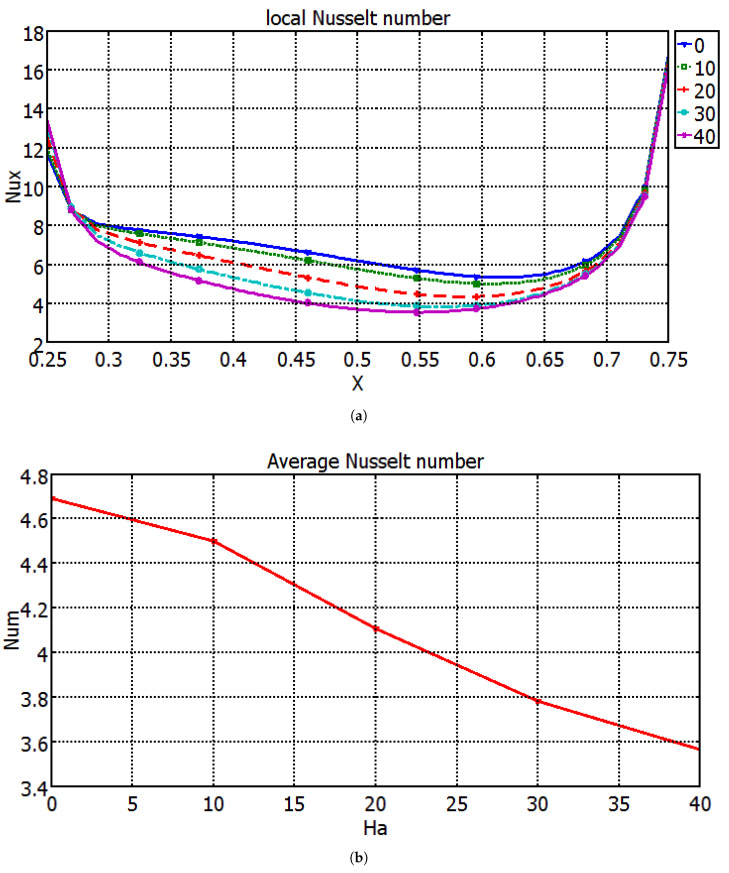
Variation of (**a**) local and (**b**) average Nusselt number along the hot bottom wall for different Hartmann numbers with the M3 model (Ri = 1, ϕ=0.02).

Influence of Hartmann number on variation of flow and thermal patterns are demonstrated in Figure 5 with M3 electrical conductivity model at Richardson number of 1. The triangular enclosure is filled with a single vortex in the absence of magnetic field. As the value of the magnetic field strength increases, the number of vortices is increased. The value of the maximum stream function also reduces, due to the dampening of the fluid motion for higher values of magnetic field strength. Isotherms become less dense, especially in the middle of the heater for higher Hartmann number values. Local and average Nusselt number decrease when the value of Hartmann number rises. It is attributed to the reduction of convection with magnetic field (Figure 6). This feature of reduction in the convective heat transfer with magnetic field in cavities was also found in many studies [45,46]. In most of the studies with MHD application, electrical conductivity based on Maxwell model was utilized. Effects of different electrical conductivity models (M1, M2 and M3) on the variation of the average Nusselt number for different Richardson number and for different Hartmann numbers are demonstrated in Table 4 and Table 5. Average heat transfer values are highest for Maxwell model as compared to other models for the same Ri and Ha values. The discrepancy between different models for average Nusselt number becomes higher as the value of the Hartmann number enhances. The value of Nusselt number reduces by about 0.98% and 3.97% for Ha = 10, whereas these values become 10.512% and 22.45% for M2 and M3 models as compared to Maxwell model. There are differences in the average heat transfer values for the same values of Hartmann number and Richardson number when different electrical conductivity models are used. As it is mentioned in Ref. [25], the Maxwell model under-predicts the electrical conductivity of nanofluid. Maxwell model is better suited for dispersions with larger particle sizes. The rate of reduction is higher for configurations with electrical conductivity models of M2 and M3 as compared to M1 since the Maxwell model underestimates nanofluid-mixture electrical conductivity.

**Figure 7:**
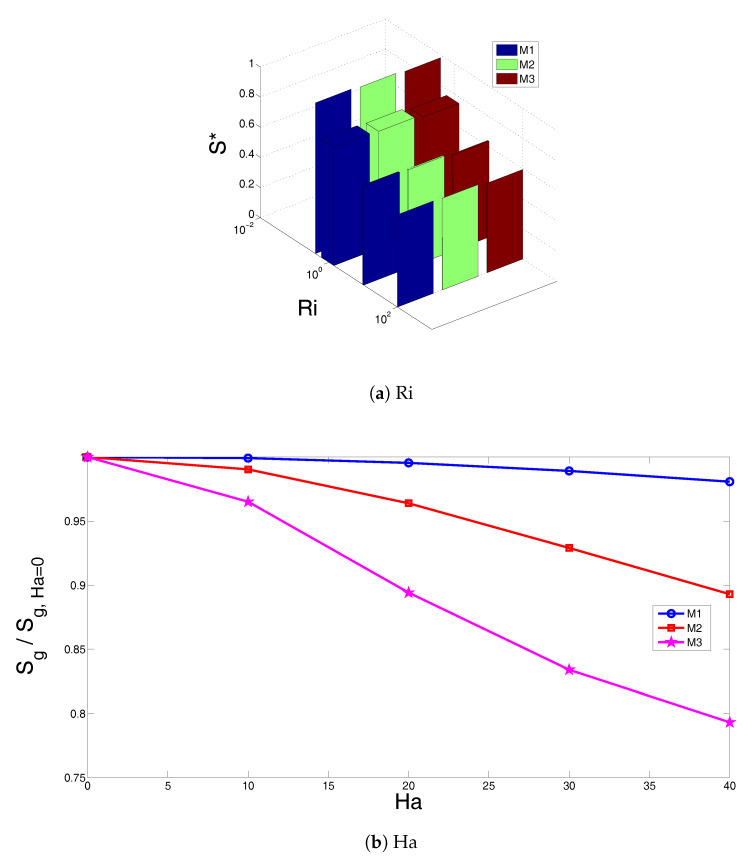
Normalized entropy generation rate versus Richardson number (Ha = 20) (**a**), and versus Hartmann number (Ri = 1) (**b**) with various electrical conductivity models of nanofluid at ϕ=0.02.

**Figure 8:**
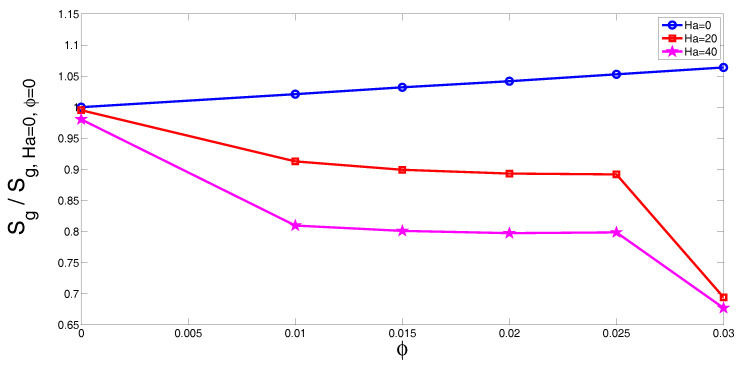
Normalized entropy generation rate versus solid particle volume fraction with model M3 for various values of Hartmann numbers (Ri = 1).

The total entropy generation of the system was also examined for different electrical conductivity models of the nanofluid. The total entropy generation values which are normalized with the values at Richardson number of 0.01 (denoted by S*) versus Richardson number are shown in Figure 7a for various models (M1, M2 and M3) at the fixed values of (Ha = 20, ϕ=0.01). The entropy generation decreases with increasing values of Richardson number and this may be attributed to the reduction of heat transfer irreversibility. In this case, there are only slight changes between different configurations with various electrical conductivity models. The total entropy generation which is normalized with respect to values at Ha = 0 versus Hartmann number is shown in Figure 7b for models M1, M2 and M3. The entropy generation rate reduces with Hartmann number and this could be attributed to the reduction of convective heat transfer and heat transfer irreversibility. The reduction rate is higher for model M3 while it is lowest for the Maxwell model. This behavior was also demonstrated in the average heat transfer variation as it was shown above. The Maxwell model underestimates the electrical conductivity of the alumina-water nanofluid mixture as it was shown in the experimental study of [25]. Therefore, more dampening of the fluid motion is expected for other models (M2 and M3) as compared to the Maxwell model when the value of the Hartmann number is the same. Figure 8 shows the variation of total entropy generation versus solid particle volume fraction for various values of Hartmann numbers. Significant changes in the entropy generation rate are seen for the configuration with M3 electrical conductivity model as compared to models M1 and M2. The entropy generation in Figure 8 is normalized with respect to values for water in the absence of magnetic field (Ha = 0, ϕ=0). In the absence of magnetic field (Ha = 0), the normalized entropy generation increases with the solid particle volume fraction. This is due to the increment in the fluid friction and heat transfer irreversibility for higher solid particle volume fractions. In the presence of magnetic field, entropy generation is first reduced up to solid particle volume fraction of ϕ=0.01, and then remains constant between 0.01≤ϕ≤0.025. At the highest particle volume fraction, a sharp reduction is seen for Ha = 20 and Ha = 40. When the nanoparticles are included in the base fluid, both the thermal conductivity and the electrical conductivity of the nanofluid change. As the Maxwell model underestimates the electrical conductivity of the nanofluid, less reduction in the heat transfer is obtained. This leads to higher heat transfer irreversibility for model M3. However, in the absence of the magnetic field, the normalized entropy generation increases with higher values of solid nanoparticle volume fraction since the fluid friction irreversibility enhances with higher ϕ values.

## 4. Conclusions

Mixed convection and entropy generation of alumina-water nanofluid in a lid driven triangular cavity were examined under the effect of magnetic field with various electrical conductivity models. It was observed that the average Nusselt number reduces for higher values of Richardson and Hartmann numbers. Significant variations for the average Nusselt numbers and normalized entropy generation rates were observed at higher Hartmann numbers between nanofluids with different electrical conductivity models. Among different models, Maxwell model gives the highest heat transfer rate for the same value of Hartmamn number and Richardson number. The rate of average heat transfer reduction with Hartmann number is highest for model M3 and it is lowest for the Maxwell model. The total entropy generation was found to decrease for higher values of Richardson number and Hartmann number. In the absence of a magnetic field, a solid particle volume fraction increment resulted in entropy generation rate enhancement, while, in the presence of a magnetic field, different behaviors were observed depending on the nanoparticle volume fraction. Transient effects, different thermal boundary conditions, various particle types and shapes may be considered along with the different electrical conductivity models of the nanofluids for future investigations.

## Figures and Tables

**Table 1:** Thermophysical properties of water and Al${}_{2}$O${}_{3}$.

Property	Water	Al${}_{2}$O${}_{3}$
ρ (kg/m${}^{3}$)	997.1	3970
$c_{p}$ (J/kg K)	4179	765
k (W m${}^{-1}$ K${}^{-1}$)	0.6	25
β (1/K)	$2.1\times 10^{-4}$	$0.85\times 10^{-5}$

**Table 2:** The average Nusselt number in Reference [40] and computed with the present solver for a lid-driven cavity problem.

Re = 400	Reference [40]	Current Solver
Gr = 100	3.84	3.81
Gr = $10^{4}$	3.62	3.63
Gr = $10^{6}$	1.22	1.26

**Table 3:** Magneto-hydrodynamics (MHD) free convection study, comparison of the average Nusselt number.

Ha	Present Study	Sheikholeslami and Shamlooei [42]	Rudraiah et al. [41]
0	2.474	2.566	2.518
10	2.172	2.266	2.223
50	1.068	1.099	1.085
100	1.009	1.022	1.011

**Table 4:** Variation of average Nusselt number along the hot wall for various electrical conductivity models and for different Richardson numbers (Ha = 10, ϕ=0.02).

Ri	M1	M2	M3
0.01	5.095	5.055	4.930
1	4.685	4.639	4.499
10	3.945	3.909	3.801
100	3.717	3.688	3.597

**Table 5:** Variation of average Nusselt number along the hot wall for various electrical conductivity models and for different Hartmann numbers (Ri = 1, ϕ=0.02).

Ha	M1	M2	M3
0	4.692	4.692	4.692
10	4.685	4.639	4.499
30	4.631	4.304	3.781
40	4.586	4.104	3.566

## Abbreviations

The following abbreviations are used in this manuscript:

**B${}_{0}$**	magnetic field strength
**Gr**	Grashof number, $\frac{g\beta_{f}(T_{h}-T_{c})H^{3}}{\nu_{f}^{2}}$
*h*	local heat transfer coefficient, (W/m${}^{2}$K)
**Ha**	Hartmann number, $B_{0}H\sqrt{\frac{\sigma_{nf}}{\rho_{nf}\nu_{f}}}$
*k*	thermal conductivity, (W/m.K)
*h*	heater size, (m)
*H*	length of the cavity, (m)
*M,1 M2, M3*	different electrical conductivity models
*n*	unit normal vector
Nu	local Nusselt number
*p*	pressure, (Pa)
*P*	non-dimensional pressure
Pr	Prandtl number, $\frac{\nu_{f}}{\alpha_{f}}$
*S**	non-dimensional entropy generation rate
*T*	temperature, (K)
*u, v*	*x*-*y* velocity components, (m/s)
*U, V*	dimensionless velocity components
*x, y*	Cartesian coordinates, (m)
*X, Y*	dimensionless coordinates
**Greek Characters**	
α	thermal diffusivity, (m${}^{2}$/s)
β	expansion coefficient, (1/K)
ϕ	nanoparticle volume fraction
θ	non-dimensional temperature, $\frac{T-T_{c}}{T_{h}-T_{c}}$
ν	kinematic viscosity, (m${}^{2}$/s)
ρ	density of the fluid, (kg/m${}^{3}$)
σ	electrical conductivity, (S/m)
**Subscripts**	
*c*	cold wall
*m*	average
*h*	hot wall
